# Viewing Teens as Responsible in Family: Implications for Chinese Youth's Academic and Social Adjustment

**DOI:** 10.1111/cdev.70013

**Published:** 2025-10-02

**Authors:** Beiming Yang, Zexi Zhou, Varun Devakonda, Bin‐Bin Chen, Yang Qu

**Affiliations:** ^1^ School of Education and Social Policy Northwestern University Evanston USA; ^2^ Department of Human Development and Family Sciences The University of Texas at Austin Austin USA; ^3^ Department of Psychology Fudan University Shanghai China

**Keywords:** academic development, adolescence, attachement, family obligation, stereotypes of teens

## Abstract

Using three‐wave longitudinal data of 554 Chinese youth (mean age = 13.35 years; 50% girls; T1 = July 2020, T2 = January 2021, T3 = July 2021), this study examined how youth's views of teens regarding family obligation predict their academic functioning and relationship with parents, with attention to the mediating role of youth's sense of responsibility to parents. Results showed that views of teens regarding family obligation predicted youth's greater academic delay of gratification, motivational response to academic failure, and attachment security to mother and father over time. Importantly, youth's sense of responsibility to parents mediated the longitudinal associations between views of teens and their academic and social adjustment. Taken together, the findings elucidate why and how views of teens matter for positive youth development in a culturally sensitive manner.

The general public in Western cultures largely views teens in a negative light, such that teens are viewed as moody, irresponsible, and rebellious (Buchanan and Bruton 2016; Buchanan and Holmbeck 1998). However, although it is true that adolescents generally experience more mood disruptions and take more risks than younger children (Arnett 1999), most adolescents do not experience severe “storm and stress” (Arnett 1999; Hollenstein and Lougheed 2013). From a lifespan perspective, challenges and crises are not unique to adolescence, given that all life stages experience their own difficulties through growth and decline (Baltes et al. 1999). Importantly, developmental changes that were considered “storm and stress” during adolescence, such as increased risk taking, can be adaptive processes for adolescents to gain the experience necessary for their growth (Romer et al. 2017; Telzer et al. 2022). Therefore, there is a strong need to characterize adolescence in a more positive light (e.g., a window of opportunity) to better support and promote positive youth development (Buchanan et al. 2023; Lerner 2017; Lerner et al. 2005; Shek et al. 2019).

Cultural variations in views of teens have shown that teen years can be viewed in a positive light in certain cultures (Qu 2023; Whiteman and Buchanan 2002). For example, teen years in China are largely viewed as a time of becoming responsible and fulfilling family obligation (Pomerantz et al. 2011; Qu et al. 2016). Scholars have suggested that fulfilling family obligation represents an important marker of maturity, as adolescents increasingly contribute to their families through their improved skills and capacities (Fuligni and Pedersen 2002). This is particularly relevant for Chinese adolescents, as fulfilling family obligation is regarded as a key virtue in Chinese culture and remains prevalent in contemporary China (Yang et al. 2024; Yeh et al. 2013). Past studies have shown that viewing the teen years as a time of fulfilling family obligation is linked with youth's positive academic development over time (e.g., greater school engagement and self‐regulated learning strategies; Qu et al. 2020, 2022). However, the influences of these beliefs on how youth respond to difficult academic situations and how they handle relationships with parents are less studied. More importantly, no study to date has examined the mechanisms through which the views of teens may contribute to positive youth development. Therefore, using a three‐wave longitudinal design, the current study examined how views of teens regarding family obligation play a role in Chinese youth's response to difficult academic situations and attachment security to parents, with attention to youth's sense of responsibility to parents as a mediator.

When youth believe teens are typically responsible in the family, these category‐based beliefs (i.e., beliefs about a broad social category) may influence the standards and expectations that youth set for themselves (Whiteman and Buchanan 2002). These personal standards, known as target‐based expectations (i.e., expectations for specific individuals, such as oneself), are manifested in their increased sense of responsibility to parents over time. Past studies suggest that the perceptions of social norms tend to influence individuals' actual behaviors by shaping the standards they set for themselves (e.g., Helms et al. 2014; Paluck and Shepherd 2012; Zou et al. 2009). Research on teen stereotypes suggests that the “storm and stress” views of teens shape teens' expectations for themselves, which can be self‐fulfilling and thus contribute to youth's unconstructive behavior as well as worse relationships with parents (Buchanan and Hughes 2009; Meece et al. 1990). For example, research on adolescent drinking suggests that parents' beliefs about adolescent drinking contribute to children's expectations of their own drinking behavior in the future, which, in turn, predicts their actual drinking behavior over adolescence (Madon et al. 2008). In the same vein, when youth believe teens are typically responsible in the family, they may set expectations and standards for themselves that are in line with such views of teens. As a result, these youth may become increasingly responsible to their parents (e.g., respect parents, help with family chores, and be willing to assist parents both emotionally and financially in the future).

In turn, youth's greater sense of responsibility to parents may play a positive role in their academic functioning and relationships with parents. Given that Chinese culture places a strong emphasis on learning (Li 2016), youth may consider academic achievement as a means to fulfill their responsibility to parents. Indeed, Chinese youth's sense of family responsibility is longitudinally associated with better academic functioning, such as greater school engagement and self‐regulated learning strategies (Fuligni 2001; Pomerantz et al. 2011; Qu and Pomerantz 2015). However, it is less clear how youth's sense of responsibility to parents can play a role in their response to academic dilemmas and challenges. For example, although youth understand the importance of academic work, they also find it less enjoyable compared to other activities (e.g., socializing with friends, playing sports, or resting; Duckworth et al. 2019), and thus it requires youth to process the ability to resist the temptation of more enjoyable activities to focus on the more important academic work (i.e., academic delay of gratification, Bembenutty and Karabenick 1998). Moreover, youth often encounter academic failures such as poor grades. In this case, it is crucial for youth to stay motivated in response to these academic failures (i.e., motivational response to academic failure), which can help them achieve long‐term academic success (Mega et al. 2014). Given the strong emphasis on academic success in Chinese culture (Li 2016), youth who feel a greater sense of responsibility to their parents may be more likely to stay motivated in difficult academic situations. This sense of responsibility enables them to prioritize academic commitments over short‐term desires and remain resilient in the face of academic challenges or failures.

Moreover, youth's greater sense of responsibility to parents may promote a closer relationship with parents (Campos et al. 2014). Past research has documented that youth who expect distancing from parents are more likely to have less closeness and more conflict with parents (Buchanan and Hughes 2009). In contrast to these negative expectations, when youth believe they should fulfill responsibilities to parents, these standards and expectations they set for themselves can help them maintain strong connectedness with parents. Such connectedness to parents provides youth the opportunity to maintain a strong emotional bond with parents, and thus parents can still work as their “secure base” as they navigate the teen years (Allen et al. 2003; Allen and Land 1999). Indeed, a prior study on familism suggests that the more youth value family connectedness, the more they are attached to their parents (Li et al. 2016). Moreover, Chinese youth's sense of responsibility toward their parents is, in part, characterized by showing respect and valuing parents' advice (e.g., following parents' guidance when choosing friends; Fuligni et al. 1999; Pomerantz et al. 2011). In this case, the more youth feel responsible to their parents, the more likely they may trust their parents as a source for advice, which will foster their secure relationship with parents. Taken together, youth's sense of responsibility may predict their greater attachment security to parents over time.

Although past research has documented that viewing adolescence as a time of fulfilling family obligation—a popular view among Chinese youth—contributes to youth's greater school engagement and self‐regulated learning strategies (Qu et al. 2016, 2022), its role in other aspects of youth development (e.g., youth's response to academic challenges and relationship with parents) and the underlying mechanisms of such impacts remain unclear. To address these gaps, the current research employed a three‐wave longitudinal design to examine whether views of teens regarding family obligation play a role in Chinese youth's academic functioning and relationship with parents, with attention to the mediating role of youth's sense of responsibility to parents. Conceptually, the current research examined how culturally shaped beliefs (i.e., views of teens in general) play a role in youth's development (i.e., academic and social adjustment) through the standard they set for themselves (i.e., how much they should fulfill responsibilities to parents). We hypothesized that views of teens regarding family obligation would predict youth's greater sense of responsibility to parents over time, which would, in turn, contribute to their improved academic functioning (i.e., academic delay of gratification and motivational response to academic failure) and relationship with parents (i.e., attachment security to mother and father) over time. Given that each component of the hypotheses, particularly the main effect of views of teens on adolescent adjustment, was well supported by the literature, this research is more confirmatory than exploratory.

## 1 Method

### 1.1 Participants

The sample consisted of 554 Chinese youth (mean age = 13.35 years, SD = 0.35; 50% girls and 50% boys). Participants were recruited from three middle schools in Shanghai. According to the schools' overall performance in the high school entrance exam, one school was above average and the other two were average achieving in Shanghai. Families were primarily from middle‐ and working‐class backgrounds. With regard to youth's parents' educational attainment, 49% of mothers and 52% of fathers had a degree beyond high school (i.e., an associate degree or above), which was similar to the local average in Shanghai at the time of data collection (Shanghai Municipal People's Government, 2021).

### 1.2 Procedure

Participants completed the same online questionnaires three times over a year, with 6 months between each wave of data collection (i.e., T1 in July 2020, T2 in January 2021, and T3 in July 2021). At each timepoint, extensive explanations of the research were given, and participants completed the online consent before taking the questionnaire. All questionnaire measures used in the current study were in Chinese (i.e., participants' native language). Ethical approval for the study was obtained from the Institutional Review Board of Fudan University. Participants received small gifts for their participation. The attrition rate from T1 to T3 was 29%. Among all the participants who completed the questionnaires at T1, 82% completed follow‐up surveys at either T2 or T3. Results in Little's MCAR test (χ^2^ (32) = 45.88, *p* = 0.053) suggested that missing cases could be missing completely at random (MCAR; Little 1988). To handle missing data, full information maximum likelihood estimation was used to provide reliable standard errors under a wide range of conditions (Schafer and Graham 2002).

### 1.3 Measures

#### 1.3.1 Views of Teens Regarding Family Obligation

At T1, youth's views of teens regarding family obligation were assessed using a measure of youth's beliefs on adolescence (Qu et al. 2016, 2020). By comparing an attitude or behavior among teens vs. younger children, this measure assesses participants' beliefs on whether teens typically fulfill more family obligation than younger children, meaning that teen years are a time of becoming responsible. For each of the 12 items on family obligation (e.g., “be a responsible member of the family” and “make sacrifices for family”), youth rated to what extent the behavior or attitude is more true before versus during the teen years on a 7‐point Likert scale (1 = *more true before teen years*, 4 = *equally true before and during teen years*, 7 = *more true during teen years*). The mean was taken across all items, with lower numbers indicating that fulfilling family obligation is viewed as more characteristic *before* the teen years and higher numbers indicating it is more characteristic *during* the teen years. This measure showed good internal consistency, with *α* = 0.84.

#### 1.3.2 Sense of Responsibility to Parents

At T1 and T2, youth's sense of responsibility to parents was assessed using the measure developed by Pomerantz et al. (2011). For each of the nine items (e.g., “respect parents” and “help parents financially when they get older”), youth indicated how much they feel they should engage in the activity described on a five‐point Likert scale (1 = *not at all* to 5 = *very much*). The mean was taken across all items, with higher numbers indicating youth's greater sense of responsibility to parents. This measure showed excellent internal consistency, with *α* = 0.94 at T1 and 0.95 at T2.

#### 1.3.3 Academic Delay of Gratification

At T1 and T3, youth completed the academic delay of gratification scale (ADOGS; Bembenutty and Karabenick 1998). Each item in this scale is an academic scenario with two options, one representing immediate gratification and one representing delayed gratification (nine items, e.g., “spend most of your time studying just the interesting material in this course even though it may mean not doing so well” vs. “study all the material that is assigned to increase your chances of doing well in the course”). For each scenario, youth indicated whether they would choose option A or B on a 4‐point Likert scale (1 = *definitely choose A*, 2 = *probably choose A*, 3 = *probably choose B*, 4 = *definitely choose B*). A total of nine scenarios were included. One scenario on course selection from the original scale was dropped because this scenario does not apply to Chinese middle school students. The mean was taken across all items, with higher numbers indicating youth's greater academic delay of gratification. This measure showed good internal consistency, with *α* = 0.82 at T1 and 0.85 at T3.

#### 1.3.4 Motivational Response to Academic Failure

At T1 and T3, youth's motivational response to academic failure was assessed with the response to failure scale in the Taxonomy of Problematic Social Situations for Children (TOPS; Dodge et al. 1985). Youth were provided with a problematic situation of receiving a poor grade on an exam. On a 5‐point Likert scale (1 = *not at all true* to 5 = *very true*), they indicated whether they would feel motivated to improve in response to academic failure (five items, e.g., “I would want to work harder in that subject” and “I would really pay attention to that subject in class”). The mean was taken across all items, with higher numbers indicating youth's greater. This measure showed excellent internal consistency, with *α* = 0.92 at T1 and 0.95 at T3.

#### 1.3.5 Attachment Security to Parents

At T1 and T3, youth's attachment security was assessed using the parental attachment measure of the Inventory of Parent and Peer Attachment (IPPA, Armsden and Greenberg 1987). On a 5‐point Likert scale (1 = *almost never or never true* to 5 = *almost always or always true*), youth reported on their attachment to mothers and fathers separately. This measure includes three broad dimensions of attachment, which are the degree of mutual trust (eight items, e.g., “I trust my mother”), quality of communication (seven items, e.g., “I tell my mother about my problems and troubles”), and extent of alienation (five items, e.g., “I get upset easily around my mother”). Scores of five items on alienation were reversed, and then the mean was taken across 20 items, with higher numbers indicating youth's greater attachment security to their parents. This measure showed good internal consistency, with *α* = 0.91 at T1 and 0.94 at T3 for attachment security to mothers and *α* = 0.91 at T1 and 0.94 at T3 for attachment security to fathers.

#### 1.3.6 Demographic Variables

Demographic characteristics, including youth's age, gender, and their parents' educational attainment, were assessed in the present study. Youth reported on their gender identity, which was coded into 0 = *boy* and 1 = *girl*. Parents' educational attainment was coded into 0 = *less than a college degree* and 1 = *college degree or higher*. The average educational attainment between mother and father was included in the analysis.

## 2 Overview of the Analyses

**FIGURE 1:**
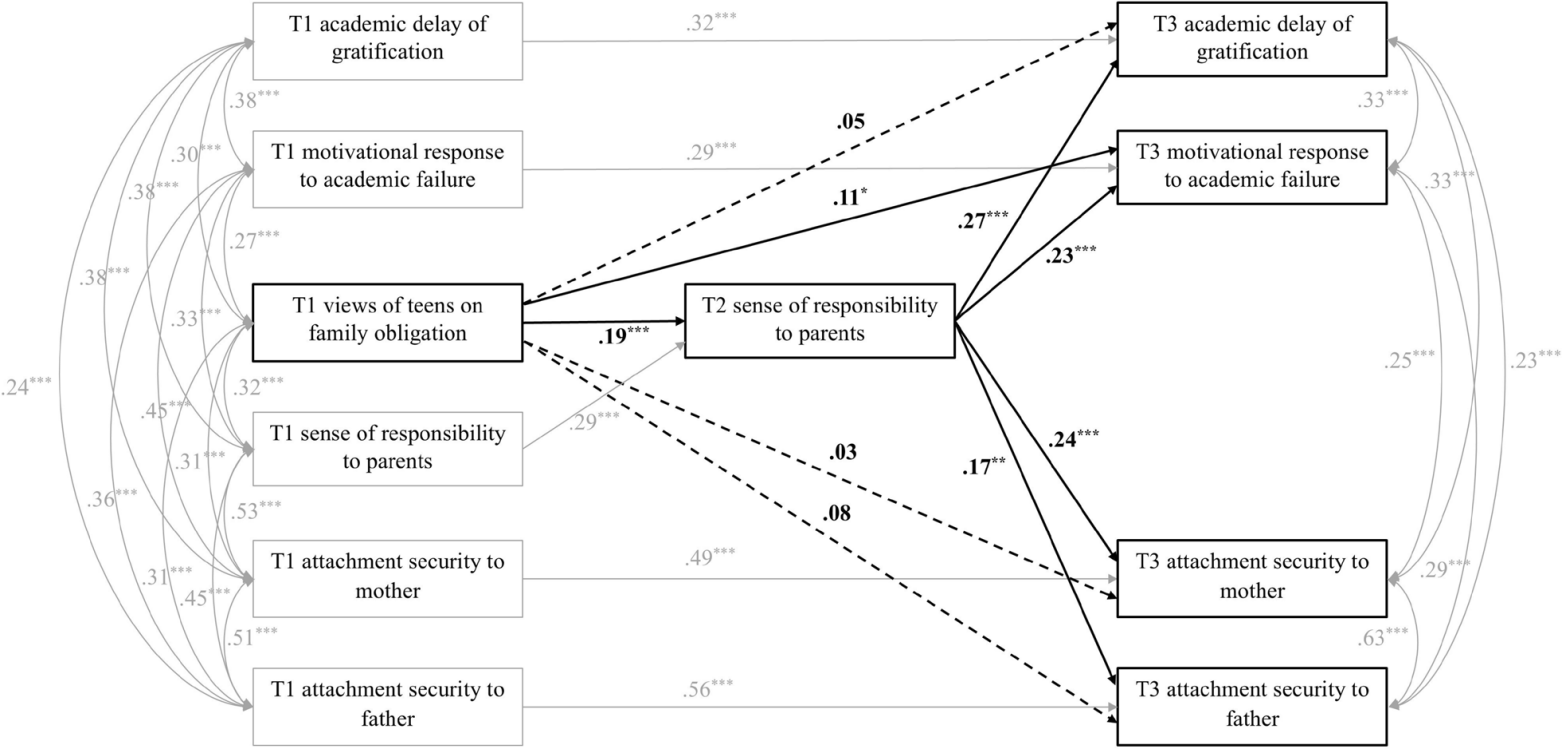
Sense of responsibility to parents mediated the effects of views of teens regarding family obligation on youth's academic delay of gratification, motivational response to academic failure, attachment security to mother, and attachment security to father. Youth's age, gender, and parents' educational attainment were included in the models as covariates but are not shown for ease of presentation. Standardized coefficients are presented. Dashed lines represent nonsignificant paths. *p < 0.05. **p < 0.01. ***p < 0.001.

Descriptives, bivariate correlations, and the main model were examined using MPlus 8.9 (Muthén and Muthén 2022). In the context of path analysis, the main analysis examined whether youth's sense of responsibility to parents mediated the longitudinal effects of youth's views of teens regarding family obligation on their academic functioning (i.e., academic delay of gratification and motivational response to academic failure) and attachment security (i.e., academic security to mother and father) over time in the same model, controlling for the initial level of each variable and demographic covariates (i.e., youth's age, youth's gender, and parents' educational attainment). As shown in Figure 1, sense of responsibility to parents at T2 was predicted by views of teens regarding family obligation at T1, controlling for sense of responsibility to parents at T1 and demographic covariates; simultaneously, youth's academic functioning and attachment security at T3 were predicted by views of teens regarding family obligation at T1 and sense of responsibility to parents at T2, controlling for the initial level of each outcome at T1 and demographic covariates. Following Finney and DiStefano's (2013) suggestion, nonnormality was handled by using maximum likelihood estimation with robust standard errors (i.e., estimator = MLR), which corrects for non‐normality–induced bias in the standard errors.

## 3 Results

### 3.1 Descriptive Statistics and Bivariate Correlations

Table 1 shows descriptive statistics and correlations between variables examined in the current study. Views of teens regarding family obligation, sense of responsibility to parents, academic delay of gratification, motivational response to academic failure, attachment security to mother, and attachment security to father were positively correlated with one another across all time points. Girls reported a lower motivational response to academic failure than boys. Youth in families with higher (vs. lower) educational attainment reported greater motivational response to academic failure and greater attachment security to both mothers and fathers.

**TABLE 1:** Descriptive statistics and correlations of variables.

	1	2	3	4	5	6	7	8	9	10	11	12	13	14
1. T1 views of teens on family obligation	—													
2. T1 sense of responsibility to parents	0.32***	—												
3. T1 academic delay of gratification	0.30***	0.38***	—											
4. T1 motivational response to failure	0.27***	0.33***	0.38***	—										
5. T1 attachment security to mother	0.31***	0.53***	0.38***	0.45***	—									
6. T1 attachment security to father	0.31***	0.45***	0.24***	0.36***	0.51***	—								
7. T2 sense of responsibility to parents	0.28***	0.35***	0.22***	0.31***	0.31***	0.30***	—							
8. T3 academic delay of gratification	0.22***	0.26***	0.42***	0.23***	0.25***	0.15**	0.35***	—						
9. T3 motivational response to failure	0.25***	0.33***	0.23***	0.39***	0.30***	0.26***	0.34***	0.44***	—					
10. T3 attachment security to mother	0.25***	0.31***	0.28***	0.31***	0.54***	0.35***	0.41***	0.45***	0.42***	—				
11. T3 attachment security to father	0.29***	0.32***	0.17**	0.28***	0.30***	0.64***	0.36***	0.30***	0.42***	0.63***	—			
12. Youth's age	0.07	−0.01	−0.05	−0.03	−0.05	0.02	−0.01	−0.05	−0.03	−0.04	−0.03	—		
13. Youth's gender	−0.02	−0.03	0.03	−0.08	0.06	0.02	−0.06	0.01	−0.12*	−0.00	−0.07	−0.04	—	
14. Parents' education	−0.02	0.04	0.04	0.09*	0.20***	0.14**	0.07	0.05	0.06	0.12*	0.11*	−0.08	−0.02	—
Mean	4.86	4.24	3.20	3.68	3.81	3.43	4.20	3.11	3.79	3.77	3.53	13.35	0.50	0.51
SD	1.15	0.80	0.55	1.04	0.86	0.90	0.79	0.61	1.03	0.82	0.84	0.35	0.50	0.46
Min	1.00	1.00	1.11	1.00	1.00	1.00	1.56	1.00	1.00	1.00	1.00	12.27	0	0
Max	7.00	5.00	4.00	5.00	5.00	5.00	5.00	4.00	5.00	5.00	5.00	15.45	1	1

*Note:* For gender, 0 = boy and 1 = girl. For parental education, 0 = less than a college degree and 1 = college degree or higher.

*p < 0.05. **p < 0.01. ***p < 0.001.

### 3.2 How Views of Teens Regarding Family Obligation Predict Youth's Academic and Social Adjustment Over Time

As shown in Figure 1, a path model was estimated to examine whether youth's (1) academic delay of gratification, (2) motivational response to academic failure, (3) attachment security to mother, and (4) attachment security to father at T3 were predicted by their views of teens regarding family obligation at T1 through their sense of responsibility to parents at T2, controlling for their adjustment at T1 and demographic covariates. This model showed good model fit, with CFI = 0.98, TLI = 0.95, RMSEA = 0.04, and SRMR = 0.03. Regarding the total effects of views of teens on adolescent adjustment, results showed that youth's views of teens regarding family obligation were associated with their greater academic delay of gratification (β = 0.10, *p* = 0.04), motivational response to academic failure (β = 0.15, *p* = 0.002), and attachment security to father (β = 0.11, *p* = 0.02), but not attachment security to mother (β = 0.07, *p* = 0.12) over one year during adolescence, adjusting for youth's prior adjustment and demographic covariates.

### 3.3 The Mediating Role of Youth's Sense of Responsibility to Parents

Regarding the mediating role of youth's sense of responsibility to parents, results showed that youth's views of teens regarding family obligation at T1 were associated with their greater sense of responsibility to parents at T2 (β = 0.19, *p* < 0.001), controlling for sense of responsibility to parents at T1 and demographic covariates; in turn, youth's sense of responsibility to parents at T2 was associated with their greater academic delay of gratification (β = 0.27, *p* < 0.001), motivational response to academic failure (β = 0.23, *p* < 0.001), attachment security to mother (β = 0.24, *p* < 0.001), and attachment security to father (β = 0.17, *p* = 0.001) at T3, adjusting for youth's adjustment at T1 and demographic covariates. Results on indirect effects showed that sense of responsibility to parents fully mediated the link between views of teens and academic delay of gratification (indirect effect: β = 0.05, *p* = 0.01, 50% reduction in the total effect), partially mediated the link between views of teens and motivational response to academic failure (indirect effect: β = 0.04, *p* = 0.009, 29% reduction in the total effect), fully mediated the link between views of teens and attachment security to mother (indirect effect: β = 0.05, *p* = 0.01, 63% reduction in the total effect), and fully mediated the link between views of teens and attachment security to father (indirect effect: β = 0.03, *p* = 0.01, 30% reduction in the total effect).

## 4 Discussion

The current study found that viewing the teen years as a time of fulfilling family obligation was associated with better response to academic challenges and greater attachment security to parents among Chinese youth over one year during midadolescence. The findings on academic functioning are consistent with prior studies that showed the positive role of views of teens regarding family obligation in youth's greater school engagement, values of school, and self‐regulated learning strategies (Qu et al. 2016, 2020, 2022). Whereas prior studies mainly focused on assessments of youth's daily learning behaviors (e.g., school engagement and learning strategy), the current research provides additional evidence on how youth's views of teens predict their response to academic challenges. Moreover, the findings on attachment security provide the first evidence of the link between views of teens regarding family obligation and youth's social adjustment. During adolescence, attachment security to parents still plays a significant protective role in almost all aspects of youth's adjustment (Allen et al. 2003; Coulombe and Yates 2022; Galbally et al. 2020; Yang et al. 2022). By adding positive social outcomes to the existing literature on views of teens regarding family obligation, the present findings help provide a more comprehensive understanding of what these responsible images entail. Echoing the call for a more positive characterization of adolescence (Buchanan et al. 2023), our findings point to the importance of characterizing adolescence as a time of becoming responsible, which can help promote positive images of teens at a societal level.

Importantly, youth's sense of responsibility to parents mediated the longitudinal associations between views of teens regarding family obligation and youth's academic and social adjustment. First, cultural beliefs on how teens typically behave shape youth's self‐expectations of how much they should respect and assist their parents. This is consistent with past research on how youth's negative views of teens (e.g., storm and stress views) can be self‐fulfilling (Buchanan and Hughes 2009; Meece et al. 1990). Then, in line with prior literature (e.g., Fuligni 2001; Pomerantz et al. 2011), this sense of responsibility to parents motivates youth to work harder in school and stay close to parents at home, which fosters their academic and social adjustment over time. Moving beyond prior research showing how views of teens regarding family obligation may play a role in adolescent development, the current study is the first to elucidate the reasons behind such impacts by demonstrating youth's sense of responsibility to parents as an underlying mechanism.

The current study has a few limitations that point to future directions. First, although the three‐wave longitudinal design is a strength of this study, the findings were based on correlational data and thus do not allow for causal conclusions. Second, the current study only followed middle school students over a year. It is crucial for future research to focus on younger age groups (e.g., pre‐ and early adolescents) for a longer time to elucidate how views of teens play a role when youth first enter adolescence. Third, the current study only focused on the role of sense of responsibility in youth's adjustment, while youth's adjustment may also contribute to their sense of responsibility to parents. Future studies can examine these potential bidirectional relations to elucidate how the sense of responsibility to parents matters for youth. Finally, the generalization of the findings to other cultures should be taken with caution. Given the notable cultural variations in views of teens, it is key for future research to examine whether views of teens play a similar role across cultural contexts, particularly in cultures that may view adolescence in a more negative light.

Viewing the teen years as a time of fulfilling family obligation has been highlighted as a protective factor of Chinese youth's adjustment. The current study is the first to examine the underlying mechanism between views of teens and youth's adjustment with a rigorous longitudinal approach. Using a three‐wave longitudinal sample of Chinese middle school students, the current study found that sense of responsibility to parents mediated the positive effects of views of teens regarding family obligation on youth's academic functioning and relationships with parents. The findings expand prior research by elucidating why and how views of teens are important for positive youth development.

## Data Availability

The data included in this study are not publicly accessible. The analytic code and the materials necessary to attempt to replicate the findings are available from the corresponding author upon reasonable request.
